# Exploring Chemical Composition, Antioxidant, Enzyme Inhibitory and Cytotoxic Properties of *Glaucium acutidentatum* Hausskn. & Bornm. from Turkey Flora: A Novel Source of Bioactive Agents to Design Functional Applications

**DOI:** 10.3390/antiox13060643

**Published:** 2024-05-25

**Authors:** Sakina Yagi, Gokhan Zengin, Abdullahi Ibrahim Uba, Magdalena Maciejewska-Turska, Elwira Sieniawska, Łukasz Świątek, Barbara Rajtar, Muammer Bahşi, Osman Guler, Stefano Dall’Acqua, Małgorzata Polz-Dacewicz

**Affiliations:** 1 Department of Botany, Faculty of Science, University of Khartoum, Khartoum 11115, Sudan; sakinayagi@gmail.com; 2 LAE, INRAE, Université de Lorraine, F-54000 Nancy, France; 3 Physiology and Biochemistry Laboratory, Department of Biology, Science Faculty, Selcuk University, Konya 42130, Turkey; gokhanzengin@selcuk.edu.tr; 4 Department of Molecular Biology and Genetics, Istanbul AREL University, Istanbul 34537, Turkey; abdullahi.iu2@gmail.com; 5 Department of Pharmacognosy with Medicinal Plant Garden, Medical University of Lublin, 20-093 Lublin, Poland; magdalena.maciejewska@umlub.pl; 6 Department of Natural Products Chemistry, Medical University of Lublin, 20-093 Lublin, Poland; esieniawska@pharmacognosy.org; 7 Department of Virology with Viral Diagnostics Laboratory, Medical University of Lublin, 20-093 Lublin, Poland; barbara.rajtar@umlub.pl (B.R.); malgorzata.polz-dacewicz@umlub.pl (M.P.-D.); 8 Department of Primary Education, Faculty of Education, Fırat University, Elazıg 23119, Turkey; muammerbahsi@gmail.com; 9 Pertek Sakine Genç Vocational School, Munzur University, Tunceli 62500, Turkey; osmanguler@munzur.edu.tr; 10 Department of Pharmaceutical and Pharmacological Sciences, University of Padova, 35131 Padua, Italy; stefano.dallacqua@unipd.it

**Keywords:** *Glaucium acutidentatum*, alkaloids, phenols, antioxidant, enzyme inhibition, cytotoxicity

## Abstract

The present study was performed to determine the chemical constituents, cytotoxicity, antioxidant and enzyme inhibition activities of the aerial parts of *Glaucium acutidentatum* Hausskn. and Bornm. (family Papaveraceae). Methanolic and aqueous extracts were prepared by maceration, homogenizer-assisted extraction (HAE) and infusion. Results showed that the highest total phenolic and flavonoids contents were obtained from the methanol extracts obtained by HAE (53.22 ± 0.10 mg GAE/g) and maceration (30.28 ± 0.51 mg RE/g), respectively. The aporphine, beznyltetrahydroisoquinoline, and protopine types of *Glaucium* alkaloids have been tentatively identified. Among them, glaucine was identified in all extracts. Flavonoids, phenolic acids, coumarins, organic acids and fatty acids were also detected. Methanolic extract obtained using the HAE method displayed the highest anti-DPPH (41.42 ± 0.62 mg TE/g), total antioxidant (1.20 ± 0.17 mmol TE/g), Cu^2+^ (113.55 ± 6.44 mg TE/g), and Fe^3+^ (74.52 ± 4.74 mg TE/g) reducing properties. The aqueous extracts obtained by infusion and HAE methods exerted the best anti-ABTS (103.59 ± 1.49 mg TE/g) and chelating (19.81 ± 0.05 mg EDTAE/g) activities, respectively. Methanolic extract from HAE recorded the highest acetylcholinesterase (2.55 ± 0.10 mg GALAE/g) and α-amylase (0.51 ± 0.02 mmol ACAE/g) inhibition activities, while that obtained by maceration showed the best butyrylcholinesterase (3.76 ± 0.31 mg GALAE/g) inhibition activity. Both extracts revealed the best tyrosinase inhibitory activity (25.15 ± 1.00 and 26.79 ± 2.36 mg KAE/g, *p* ≥ 0.05). *G. acutidentatum* maceration-derived aqueous extract showed selective anticancer activity against cells originating from human hypopharyngeal carcinoma. In conclusion, these findings indicated that *G. acutidentatum* is a promising source of alkaloids and phenolic compounds for variable pharmaceutical formulations.

## 1. Introduction

The genus *Glaucium* Mill. (family Papaveraceae) consists of 23 species of annual, biennial, and perennial flowering plants distributed mainly in Europe, North America, and southwest Asia [1]. Iran, followed by Turkey, is the richest country, with 17 and 12 *Glaucium* species, respectively [2]. They are known as horned poppies as their pods have a horn structure. In traditional medicine, many *Glaucium* species are reported to cure various ailments like headaches, eye problems, wounds, joint pain, constipation, and liver disorders [3,4]. Due to their richness in alkaloids, they are used as narcotics and hypnotics and most of their pharmacological activities are associated with the nervous system [4]. However, other biological activities like anticancer [5], antimicrobial [6], antioxidant [7], and antidiabetics [8] activities have also been reported. The major classes of alkaloids identified in *Glaucium* species are isoquinolines, including aporphines, benzylisoquinolines, and protopines, as well as benzophenanthridines, orphinanes, and protoberberine. Glaucine, first isolated from *G. flavum*, is a cough suppressant [9], and besides its antitussive effect, it has been shown to possess anticancer activity [10]. A detailed description of the phytoconstituents and pharmacology of *Glaucium* species has been outlined by Akaberi, et al. [11].

Among the 12 *Glaucium* species reported in the flora of Turkey, 7 are endemic [2]. Traditionally, they have been used as antitussive, analgesic, narcotic, sedative, and antihemorrhoidal substances and in the treatment of skin disorders [12,13,14,15]. Some studies on *Glaucium* species from Turkey were also performed. For example, six isoquinoline alkaloids, glaucine, isocorydine, protopine, cryptopine, allocryptopine and trans-canadine methochloride have been obtained from the aerial parts of *G. grandiflorum* [16]. Allocryptopine, protopine, berbithine and reticuline were obtained from *G. grandiflorum* var. *grandiflorum* and *G. corniculatum* [17,18]. A novel compound, glauciumoline, in addition to seven known isoquinolines, was isolated from the aerial parts of *G. corniculatum* var. *corniculatum* and *G. grandiflorum* subsp. *refractum* var. *torquatum* [7]. The biological activity, including antioxidant, anti-acetylcholinesterase, anti-inflammatory, antimicrobial, and anticancer activities of *Glaucium* species grown in Turkey were also demonstrated [7,19]. However, one of the less studied species is *G. acutidentatum* Hausskn. and Bornm. Only one report on the total alkaloids, phenolics, and flavonoids contents, as well as its acetylcholinesterase and antiproliferative activities, was found [4]. Thus, the present study was performed to determine the chemical constituents of the aerial parts of *G. acutidentatum* using different extraction methods. Additionally, the cytotoxicity and antioxidant activity of different extracts based on their capacity to scavenge free radicals, chelate, and reduce metal ions were evaluated. Their ability to inhibit enzymes implicated in diabetes, skin hyperpigmentation, and Alzheimer’s diseases was also evaluated.

## 2. Materials and Methods

### 2.1. Plant Material

Plant materials were gathered from a field investigation in 2022 (Elazığ, between Harput and Elazığ). Taxonomic identification, performed by Dr. Ugur Cakilcioglu, resulted in the deposition of a specimen in the herbarium of Munzur University (Voucher No: UC-20-17). The field study on plants (either cultivated or wild), including the collection of plant material, was performed in accordance with relevant institutional, national, and international guidelines and legislation. Aerial parts were meticulously separated, dried in the shade at room temperature, ground into powder using a laboratory mill, and stored in darkness.

### 2.2. Extraction

Maceration (MAC) and Homogenizer assisted extraction (HAE) were performed. Two solvents, namely methanol and water, were utilized in the preparation of extracts. Maceration of each 10 g plant material with 200 mL of methanol or water was carried out overnight at room temperature. In HAE, the plant material (5 g) was extracted with solvents (100 mL) using an ultra-turrax at 6000× *g* for 5 min. Using the infusion method, the plant material (10 g) was soaked in boiled water for 15 min to obtain the water extract. Subsequently, the organic solvents were evaporated for solvent removal, and the resulting water extracts were dried using a freeze-dryer.

### 2.3. Assay for Total Phenolic and Flavonoid Contents

The quantification of phenols and flavonoids was conducted in accordance with the procedures outlined in the earlier methodologies and their details are given in Supplemental Materials [20,21]. The results are expressed as gallic acid equivalents (GAE) and rutin equivalents (RE), respectively.

### 2.4. Liquid Chromatography—Mass Spectrometry Analysis

The phytochemical analysis was conducted using Agilent 1200 Infinity HPLC coupled to Agilent 6530B QTOF system (Agilent Technologies, Santa Clara, CA, USA). The extracts (10 μL) were separated on C18 Gemini^®^ column (3 μm i.d. with TMS end-capping, 110 Å, 100 × 2 mm) supported by a guard column (Phenomenex Inc., Torrance, CA, USA) by the following gradient system: 0–60% B for 45 min, 60–95% B for 1 min, and 95% B for 9 min; A was water with 0.1% formic acid *v*/*v*, while B acetonitrile with 0.1% formic acid *v*/*v*. The flow rate was maintained at 0.2 mL/min at 20 °C. Positive and negative ions generated in ESI ion source (nebulizer pressure: 35 psig, drying gas temp: 275 °C, drying gas flow: 10 L/min) were fragmented at the collision energies of 10 and 30 eV and detected in a range of 50–1700 *m*/*z*. Other working parameters were as follows: sheath gas temp: 325 °C, sheath gas flow: 12 L/min; skimmer 65 V, capillary V (+): 4000 V, and fragmentor 140 V. The identification was based on accurate masses and fragmentation patterns, also supported by available literature sources [22].

### 2.5. Antioxidant Tests

In vitro antioxidant assays, based on previously reported techniques, were executed. The 2,2-diphenyl-1-picrylhydrazyl (DPPH), 2,2′-azino-bis (3-ethylbenzothiazoline-6-sulfonic acid) (ABTS) radical scavenging assays were conducted using the methods described by Kirby and Schmidt [23] and Re et al. [24]. As reducing power assays, cupric-reducing antioxidant capacity (CUPRAC) and ferric-reducing antioxidant power (FRAP) tests were performed using the methods of Apak et al. [25] and Benzie and Strain [26]. Based on the method of Prieto et al. [27], the antioxidant potential assessed by the phosphomolybdenum (PBD) assay was measured. The results of the above assays were expressed as Trolox equivalents (TE). As another antioxidant assay, the metal chelating (MCA) test was performed as described by Dinis et al. [28] and the results were expressed as mg of disodium edetate equivalents (EDTAE) per gram of extract. All details of the methods are given in Supplemental Materials.

### 2.6. Enzyme Inhibitory Tests

Enzyme inhibition experiments were performed on the samples in accordance with established protocols. The quantification of amylase and glucosidase activity inhibition was expressed as mmol of acarbose equivalents (ACAE) per gram of extract, the assays were performed as described by Safasik [29] and Ting et al. [30], respectively. The acetylcholinesterase (AChE) and butyrylcholinesterase (BChE) activity inhibition assays were applied, based on Elmann’s method [31], and the results are expressed as mg of galanthamine equivalents (GALAE) per gram of extract. Tyrosinase inhibition was measured as reported by Masuda et al. [32] and the results were evaluated as mg of kojic acid equivalents (KAE) per gram of the tested extracts. All details of the methods are given in Supplemental Materials.

### 2.7. Cytotoxic Evaluation

Cytotoxicity was tested using an MTT (3-(4,5-dimethylthiazol-2-yl)-2,5-diphenyltetrazolium bromide)-based assay according to the previously described methodology [33]. Cells were acquired from the American Type Culture Collection (ATCC) and included non-cancerous VERO cells as well as cancer-derived cells, namely AGS (ATCC: CRL-1739, human gastric adenocarcinoma), FaDu (ATCC: HTB-43, human hypopharyngeal squamous cell carcinoma), and RKO (ATCC: CRL-2577, human colon cancer). To obtain stock solutions for biological studies, the extracts obtained using methanol were dissolved (50 mg/mL) in DMSO, while aqueous extracts were dissolved in phosphate-buffered saline. Briefly, the monolayer of the appropriate cell line was incubated with tested extracts diluted in cell media for 72 h, and then cellular viability was assessed using the MTT method. Absorbance was measured (540 and 620 nm) using the Synergy H1 Multi-Mode Microplate Reader (BioTek Instruments, Inc., Winooski, VY, USA) with Gen5 software (ver. 3.09.07; BioTek Instruments, Inc.) and the results were further analyzed using GraphPad Prism software (ver. 9.0.0, GraphPad Software, Boston, MA, USA). The CC_50_ (the 50% cytotoxic concentration; concentration resulting in a 50% reduction of cell viability) values were calculated from dose–response curves (non-linear regression). Moreover, the selectivity toward cancer cells was assessed by calculating the selectivity indexes (VERO CC_50_/cancer cell line CC_50_). Differences in CC_50_ values between cell lines were statistically analyzed using GraphPad Prism (two-way ANOVA, Tukey’s multiple comparisons test).

### 2.8. Molecular Modeling

These are the proteins’ X-ray crystal structures that were retrieved from the Protein Data Bank (https://www.rcsb.org/) [34]: α-amylase (PDB ID: 1B2Y) [35], AChE (PDB ID: 6O52) [36], BChE (PDB ID: 6EQP) [37], “CDK2 in complex with inhibitor RC-3-89” (PDB ID: 4GCJ) [38], and “Factor Inhibiting HIF (FIH) in complex with zinc and GSK128863” (PDB ID: 5OP6). Furthermore, homology models of human glucosidase and tyrosinase were retrieved from our previous study [39]. With the help of playmolecule’s “prepareProtein” server (https://www.playmolecule.com/ accessed on 2 February 2024), all proteins were prepared using the estimated pKa values of the titratable residues in each protein [40]. In general, members of the genus *Glaucium* are known to be rich in alkaloids, and thus, we focused on the interactions between alkaloids and the selected targets. All compounds’ 3D structures were obtained from the PubChem database (https://pubchem.ncbi.nlm.nih.gov/ accessed on 2 February 2024) and optimized using the UCSF Chimera tool [41]. Docking grid files were made utilizing the cocrystal ligand binding coordinates with the help of the MGLTools 1.5.6 program. AChE (x: 5.01, y: 35.37, and z: −8.38 Å), BChE (x: 42.16, y: −17.91, and z: 42.72 Å), tyrosinase (x: 29.99, y: 18.21, and z: 96.45 Å), amylase (x: −1.54, y: −44.04, and z: 22.63 Å), glucosidase (x: −13.77, y: 24.04, z: 12.35 Å). For all proteins, a grid box of x: 40, y: 40, and z: 40 Å dimension was used. As a result of this procedure, every hydrogen atom was united and given a Gasteiger partial charge. To dock, AutoDock 4.2.6 (https://autodock.scripts.edu/ accessed on 2 February 2024) was used while implementing a previously described docking method [42]. Using Biovia DS Visualizer v4.5 (BIOVIA, San Diego, CA, USA), protein–ligand interaction was investigated, and docking binding energy scores in kcal/mol were computed.

### 2.9. Statistical Analysis

The experiments were executed in triplicate, and differences among the extracts were assessed using two-way ANOVA followed by Tukey’s multiple comparisons test. The statistical analysis was conducted using Graph Pad Prism (version 9.2).

## 3. Results and Discussion

Methanolic (M) and aqueous (W) extracts of aerial parts of *G. acutidentatum* were examined for their chemical composition and antioxidant, cytotoxic, and enzyme-inhibitory activities. Different extracts were coded as follows: HAE-M and HAE-W represent extracts obtained from homogenizer-assisted extraction; MAC-M and MAC-W are extracts prepared by maceration, and INF-W is the aqueous extract obtained by infusion.

### 3.1. Total Phenolic (TPC) and Flavonoids (TFC) Contents

The TPC and TFC in different extracts of *G. acutidentatum* aerial parts were determined, and the results are presented in Table 1. The TPC ranged between 23.10 and 53.22 mg GAE/g, and the content in different extracts was in the following descending order: HAE-M > MAC-M > INF-W > MAC-W > HAE-W (*p* < 0.05). The TFC was in the range of 1.32 and 30.28 mg RE/g, with the highest significant (*p* < 0.05) content recorded from MAC-M followed by HAE-M. All other extracts had low TFC (≤2.10 mg RE/g). Thus, it was clear that methanol as a solvent recovered higher phenolic substances than water, with HAE as the best method of extraction of TPC and MAC method for TFC. These results were far higher than those recorded by Kocanci, Hamamcioglu, and Aslım [4], who reported TPC values of 0.70 and 1.00 mg GAE/g and TFC values of 1.84 and 1.62 mg RE/g for the methanol and aqueous extracts, respectively. In fact, the recovery of phenolics from plant materials is affected by many factors, including genetic diversity, the age of the plant, different environmental conditions and harvesting season, as well as the type of solvent extraction and extraction process [43].

### 3.2. Chemical Profile of Extracts

In this study, a total of 55 compounds were tentatively identified in various methanolic and aqueous extracts of aerial parts of *G. acutidentatum*, using high-performance liquid chromatography (RP-HPLC) coupled with electron spray ionization-quadrupole/time of flight-mass spectrometry (ESI-QToF/MS-MS). These specialized metabolites were classified as phenolic acids (hydroxycinnamic acid derivatives), flavonoids, alkaloids, coumarins, and organic and fatty acids. Among them, alkaloids are the most extensively studied group of metabolites reported in various species of *Glaucium* spp. [11,44,45]. To our knowledge, this is the first report comparing the phytochemical profile of *G. acutidentatum* extracts prepared using traditional and modern extraction techniques. Spectrometric data acquired in negative ionization mode gave reliable results for polyphenols (flavonoids, phenolic acids, and coumarins), whereas positive ionization mode was suitable for alkaloid identification. The aporphine, beznyltetrahydroisoquinoline, and protopine types of alkaloids have been identified. Among them, glaucine (**30**) was the most abundant alkaloid identified in all samples. The MS data, including retention time, molecular formula, precursor, and product ions are summarized in Table 2.

Polyphenols were represented mainly by flavonoids, which occurred in the form of glycosides. Only two compounds were noted in methanolic extracts of *G. acutidentatum* as aglycones: quercetin (**43**) and isorhamnetin (**44**). Glycosides of quercetin, isorhamnetin, and kaempferol were characterized by both fragmentation ions at *m*/*z* 301, 314, and 285, respectively, derived from aglycone core, after neutral loss of sugar (−162 Da, −132 Da, −146 Da) moiety and diagnostic RDA fragments [46]. Among them six compounds identified as isorhamnetin 3-*O*-rutinoside (**37**), isoquercitrin (**38**), kaempferol-7-*O*-hexoside (**39**), kaempferol-3-*O*-hexoside-pentoside (**40**), quercetin-3-*O*-rhamnoside isomer (**41**), isorhamnetin-3-*O*-hexoside (**42**) were found exclusively in methanolic extracts. Rutoside (**36**) was the only one identified in all five samples.

Out of the eight phenolic acids detected in the *G. acutidentatum* extracts, three were coumaric acid derivatives (**8**, **9**, and **13**), one hydroxybenzoic acid derivative (**7**), whereas the remaining were ferulic acid derivatives (**10**, **11**, **14**). In the case of compound **12**, spotted only in methanolic extracts, the collision-induced dissociation (CID) resulted in product ions almost identical to previously published MS/MS data for caffeoylmalic acid, reported in *Trifolium pretense* L. [47]. The precursor ion [M−H]^−^ at *m*/*z* 295.0545 was in accordance with an empirical formula of C_13_H_12_O_8_. Considering the fragmentation pattern, together with UV–vis spectra obtained, compound **12** was tentatively identified as caffeoylmalic acid. Ferulic acid derivative (**10**), 2-feruoyl-isocitric acid (**11**), and feruloylmalic acid (**14**) were identified in all five samples and shared a similar fragmentation pathway. An intense fragmentation ion at *m*/*z* 193, 173, and 134, observed in their MS/MS spectra was associated with the ferulic acid molecule. According to Masike, et al. [48], the presence of characteristic product ions at *m*/*z* 155 and at *m*/*z* 111 and the lack of fragment ion at *m*/*z* 191 in MS/MS spectra, enabled the distinguishing of hydroxycinnamoyl-isocitric acids from hydroxycinnamoyl-quinic acids. Therefore, on the basis of detected fragmentation ions in negative ionization mode (see Table 2), compound **11** was proposed to be 2-feruoyl-isocitric acid while compound **9**—3-*p*-coumaroylquinic acid. The additional product ion at *m*/*z* 115.0049 observed in MS/MS spectra for compound **14** might indicate malic acid substitution, hence it was tentatively assigned as feruloylmalic acid. Besides phenolic acids, two coumarins: dihydroxycoumarin-hexoside (**34**) and dihydroxycoumarin (**35**), were spotted in the negative ionization mode of methanolic extracts.

The MS spectra of all samples, analyzed in positive ionization mode, revealed the most intensive peak for compound **30** eluted at 20.83 min. The protonated molecular ion at *m*/*z* 356.1879 supported the molecular formula of C_21_H_25_NO_4_. An intense product ion at *m*/*z* 325.1371 [M+H−31]^+^ observed in MS/MS spectra, was deducted to be formed by neutral loss of –NH_2_CH_3_ from [M+H]^+^, while fragmentation ions shown in Table 2 suggested subsequent elimination of small molecules from product ion, such as –CH_3_, –OCH_3_ and –CO, indicating the aporphine alkaloid. Spectroscopic (λ_max_ = 220, 280, 305 nm) and spectrometric data (LC-MS/MS data) acquired in this study were in accordance with data published by Sun et al. [49] and Bournine et al. [44] for glaucine, therefore, the compound was unambiguously identified as glaucine, while compound **33** as its structural isomer, for example, takatonin (PubChem). Seven compounds (**16**, **22**, **23**, **26**, **27**, **31**, **32**) detected in all samples, shared similar behavior to glaucine fragmentation and formed characteristics for aporphinoids, product ions with *m*/*z* greater than 200 Da [50]. The precursor ion [M+H]^+^ of compound **23** at *m*/*z* 342.1715 was in accordance with the empirical molecular formula of C_20_H_23_NO_4_. Compound **26** presented a similar fragmentation pattern but differed in the intensity of the generated product ions. A comparison of the product ions with the highest intensity at *m*/*z* 311.1266 for compound **23** and at *m*/*z* 279.0994 for compound **26** allowed their identification as isocorydine and corydine, respectively, reported in *G. aleppicum* by Barakat et al. (2016). Two compounds, **27** and **31**, afforded precursor ions at *m*/*z* 372 and a molecular formula of C_21_H_25_NO_5_, suggesting the glaucine structure with an additional OH group. Characteristic neutral loss of –NH_2_CH_3_ from the precursor ion of compound **31** resulted in a product ion at *m*/*z* 341.1370 and 16 Da less at *m*/*z* 325.1424 (C_20_H_20_O_4_^+^). For compound **27**, the neutral loss of H_2_O from the precursor ion at *m*/*z* 372.1832 led to the formation of an intense fragmentation ion at *m*/*z* 354.1682 (C_21_H_24_NO_4_^+^). A small product ion at *m*/*z* 323.1275 (C_20_H_18_O_4_^+^) formed as a result of –NH_2_CH_3_–H_2_O loss was also detected in its MS/MS spectrum. By comparison of the fragmentation behavior of these compounds, compound **31** was identified as cataline, while compound **27**, which differs only in the position of the OH group, as hydroxyglaucine [11,45]. Using the same approach, compound **32** with a precursor ion at *m*/*z* 352.1203 (and the following fragmentation ions at *m*/*z*: 337.0920, 336.0836, 322.0688, 307.0775) was proposed to be corunnine (glauvine), previously identified in *G. flavum* var. *vestitum* [51]. Three compounds, **16**, **22**, and **19**, generated product ions at *m*/*z* 328 (C_19_H_21_NO_4_), which was 28 Da less than that of glaucine. The fragment ions of compounds **16** and **22**, generated after CID, were almost identical and greater than 200 Da, suggesting aporphine alkaloids. However, the acquired spectrometric data were found to be insufficient for unambiguous identification of compounds **16** and **22**, and therefore, they were identified as structural isomers—isoboldine and boldine. In the case of compound **19**, the benzyltetrahydroisoquioline structure was under consideration due to being observed in the MS/MS spectrum major product ions with *m*/*z* less than 200 Da, suggesting α and/or β-cleavage of the alkaloid skeleton. Its fragmentation ions (at *m*/*z* 297.1105, 265.0840) observed in MS/MS spectra were 2 Da less than that of compound **20**, tentatively identified as reticuline (Han et al., 2010), while major product ions of high intensity (at *m*/*z* 192.1003, 175.0735, 137.0576) were almost the same as that of reticuline, suggesting 1,2-dehydroreticuline structure, identified for the first time in *G. acutidentatum* extracts (see Table 2). Compounds **20** and **25** shared similar fragmentation ions but they differed only in retention time and occurrence. Compound **20**, tentatively identified as reticuline, was detected in all five extracts, while compound **25** (reticuline isomer) was found solely in methanolic extracts. Analysis of the product ion of compound **24** (C_20_H_25_NO_4_), at *m*/*z* 189.0875 generated by β-cleavage, suggested an additional CH_3_ group in reticuline structure, while detection of the product ion at *m*/*z* 137.0575 indicated the presence of one OH and one OCH_3_ group in C-ring as in the case of reticuline. Considering the retention time (after reticuline) and fragmentation behavior described above, compound **24** was assigned as laudanine [52], while compound **18** with similar to laudanine precursor ion at *m*/*z* 344.1866 (C_20_H_25_NO_4_) and reticuline-like fragmentation pattern, was proposed to be reticuline derivate. Taking into account the biosynthetic pathway of isoquinoline alkaloids like reticuline [53,54,55], the structures of *N*-methylcoclaurine (**17**) and its derivative 4′-*O*-methyl-*N*-methylcoclaurine (**21**) were also noted in the positive ionization mode of all analyzed extracts [56]. The results of the LC/MS study revealed another compound (**15**) having a precursor ion [M+H]^+^ at *m*/*z* 314.1759 similar to 4′-*O*-methyl-*N*-methylcoclaurine, supporting the molecular formula of C_19_H_23_NO_3_. By comparing the acquired spectrometric data with those reported by Zuo et al. [50], compound **15** generated similar fragmentation ions as described for magnocurarine. The intensive product ion at *m*/*z* 297.0995 was detected as a result of neutral loss of –NH(CH_3_)_2_ from the precursor ion. Further fragmentation resulted in distinctive product ions at *m*/*z* 175.0684 and 143.0417, suggesting β-cleavage with subsequent elimination of the –CH_3_OH group, whereas the fragmentation ion at *m*/*z* 107.0421 indicated substitution with a single hydroxyl group in the C-ring as presented by Zuo et al. [50]. However, two additional product ions at *m*/*z* 143.0417 and 121.0571 with low intensities were spotted in the MS/MS spectrum, suggesting that compound **15** may be lotusine rather than magnocurarine [56], hence compound **15** was tentatively identified as lotusine. The last type of alkaloids found in *G. acutidentatum* extracts were protopine alkaloids, which are characterized by B-ring cleavage and/or RDA fragmentation with the subsequent formation of product ions below 230 Da [50]. Two peaks, 28 and 29, with precursor ions at *m*/*z* 354.1237 and 370.1624, respectively, had the same basic skeleton of protopine alkaloid. By comparing their fragmentation behavior with those reported by Barakat, et al. [57], compound **28** was unambiguously identified as protopine, while compound **29** as α-allocryptopine, identified for the first time in *G. acutidentatum*. Diagnostic ions with *m*/*z* values of 189 and 188, resulting from B-ring cleavage and/or RDA fragmentation, observed in the MS/MS spectra of both compounds, confirmed our assumption [50]. In addition, small product ions at *m*/*z* 336.1223 and 352.1520 were found to be generated by the neutral loss of H_2_O from the precursor ion of compound **28** and compound **29**, respectively, distinguishing them from tetrahydroprotoberberine and *N*-methyltetrahydroprotoberberine alkaloids [50].

**Table 2:** Chemical profile of extracts from *Glaucium acutidentatum* aerial parts.

No	Tentative Identification	Rt (min)	Molecular Formula	Precursor Ion (*m*/*z*)	Fragment Ions (*m*/*z*)	HAE-M	HAE-W	MAC-M	MAC-W	INF-W	Ref.
	**Organic acids**										
1.	Malic acid	1.85	C_4_H_6_O_5_	133.0008 ^a^	115.0037; 89.0245; 72.9956; 71.0158	√	√	√	√	√	[58]
2.	Maleic acid	2.18	C_4_H_4_O_4_	115.0058 ^a^	73.0305; **71.0154**; 87.0103	√		√	√	√	Fragmentation; PubChem
3.	Citric acid	2.26	C_6_H_8_O_7_	191.0230 ^a^	**111.0067**; 87.0079; 57.0341	√	√		√	√	[58]
4.	Succinic acid	2.86	C_4_H_6_O_4_	117.0207 ^a^	99.0101; **73.0310**; 55.0211					√	Fragmentation; PubChem
5.	Fumaric acid	3.45	C_4_H_4_O_4_	115.0040 ^a^	99.0088; **73.0298**					√	Fragmentation; Kegg
6.	Isopropylmalic acid	13.62	C_7_H_12_O_5_	175.0633 ^a^	115.0390; 113.0615; 85.0655	√	√	√	√	√	PubChem
	**Phenolic acids**										
7.	Dihydroxybenzoic acid hexoside	12.20	C_13_H_15_O_9_	315.1090 ^a^	153.0562; 135.0455; **123.0453**; 109.0289	√		√		√	Fragmentation; PubChem
8.	beta-D-Glucosyl-2-coumarate	16.26	C_15_H_18_O_8_	325.0969 ^a^	163.0376; **119.0502**	√		√		√	Fragmentation; PubChem
9.	3-*p*-Coumaroylquinic acid	16.71	C_16_H_18_O_8_	337.0932 ^a^	191.0548; **163.0397**; 119.0500	√	√	√		√	Fragmentation; PubChem
10.	Ferulic acid derivative	19.58	—	551.1859 ^a^	**193.0503**; 178.0268; 149.0609; 134.0356	√	√	√	√	√	Fragmentation; PubChem
11.	2-Feruoyl-isocitric acid	21.16	C_17_H_20_O_9_	367.1090 ^a^	193.0490; **173.0453**; 155.0343; 134.0366; 111.0448	√	√	√	√	√	[48]
12.	Caffeoylmalic acid (=Phaselic acid)	21.62	C_13_H_12_O_8_	295.0545 ^a^	179.0336; 135.0429; 134.0179; **133.0137**; 115.0040	√		√			[47]
13.	Malic acid *p*-coumarate	24.52	C_13_H_12_O_7_	279.0558 ^a^	**163.0393**; 133.0138; 119.0497	√	√	√	√	√	Fragmentation; PubChem
14.	Feruloylmalic acid	25.27	C_14_H_14_O_8_	309.0650 ^a^	**193.0511**; 178.0270; 149.0609; 134.0371; 115.0049	√	√	√	√	√	Fragmentation
	**Alkaloids**										
15.	Lotusine	13.24	C_19_H_23_NO_3_	314.1759 ^b^	**269.1086**; 237.0828; 175.0684; 143.0417; 121.0571; 107.0421	√	√	√	√	√	[56]
16.	Isoboldine or boldine	14.02	C_19_H_21_NO_4_	328.1537 ^b^	**297.0995**; 282.0759; 265.0728; 251.0570	√	√	√	√	√	[11]
17.	N-methylcoclaurine	14.79	C_18_H_21_NO_3_	300.1579 ^b^	**269.1145**; 237.0897; **175.0734**; 137.0555; 107.0467	√		√		√	[50,55]
18.	Reticuline derivative	15.16	C_20_H_25_NO_4_	344.1866 ^b^	299.1262; 267.1000; 192.1006; 175.0745; **137.0587**	√	√	√	√	√	Fragmentation
19.	1,2-dehydroreticuline	15.52	C_19_H_21_NO_4_	328.1534 ^b^	297.1105; 265.0840; **192.1003;** 175.0735; 137.0576	√	√	√	√	√	Fragmentation; PubChem
20.	Reticuline	16.11	C_19_H_23_NO_4_	330.1710 ^b^	299.1275; 267.1012; **192.1013**; 175.0745; 137.0594	√	√	√	√	√	Fragmentation; PubChem [52]
21.	4′-*O*-Methyl-N-methylcoclaurine	16.21	C_19_H_23_NO_3_	314.1759 ^b^	299.1129; **269.1153**; 175.0767; 137.0590; 107.0485	√	√	√			[53,55]
22.	Isoboldine or boldine	16.86	C_19_H_21_NO_4_	328.1542 ^b^	**297.1116**; 282.0873; 265.0857; 251.0570	√	√	√	√	√	Fragmentation; [11]
23.	Isocorydine	17.53	C_20_H_23_NO_4_	342.1715 ^b^	**311.1266**; 280.1064; 279.0997; 206.1163; 189.0746	√	√	√	√	√	[57]
24.	Laudanine	17.77	C_20_H_25_NO_4_	344.1827 ^b^	313.1390; 281.1077; **206.1152**; 189.0875; 137.0575	√		√	√	√	Fragmentation; [52]
25.	Reticuline isomer	18.29	C_19_H_23_NO_4_	330.1710 ^b^	299.1230; 267.0935; **192.1000**; 175.0736; 137.0588	√		√			Fragmentation; [52]
26.	Corydine (=glaucentrin)	18.45	C_20_H_23_NO_4_	342.1675 ^b^	311.1258; 280.1037; **279.0994**; 189.0890	√	√	√	√	√	[57]
27.	3-Hydroxyglaucine	18.68	C_21_H_25_NO_5_	372.1832 ^b^	354.1682; **323.1275**; 308.1061	√	√	√	√	√	PubChem
28.	Protopine	18.79	C_20_H_19_NO_5_	354.1327 ^b^	336.1223; 275.0678; 206.0782; **189.0762**; 188.0687; 149.0592	√	√	√	√	√	PubChem; [57]
29.	α-Allocryptopine	19.34	C_21_H_23_NO_5_	370.1624 ^b^	352.1520; 306.0887; 290.0906; 206.0783; 189.0751; **188.0706**; 181.0828; 165.0883	√	√	√	√	√	[57]
30.	Glaucine syn. Boldine dimethyl ether	20.83	C_21_H_25_NO_4_	356.1879 ^b^	325.1371; 310.1138; 295.1033; **294.1188**; 279.0962; 251.1011	√	√	√	√	√	PubChem; Fragmentation
31.	Cataline	22.45	C_21_H_25_NO_5_	372.1797 ^b^	355.1753; 341.1370; **325.1424**; 312.1342	√	√	√	√	√	[11,45]
32.	Corunnine (=glauvine)	30.19	C_20_H_18_NO_5_	352.1203 ^b^	337.0920; 336.0836; 322.0688; 307.0775; **306.0744**; 294.1212; 279.1000; 251.1025	√	√	√	√	√	[51]
33.	Glaucine isomer (e.g., takatonin)	32.06	C_21_H_25_NO_4_	356.1879 ^b^	325.1421; 310.1176; 295.1043; **294.1230**; 279.0991; 251.1047	√	√	√	√	√	Fragmentation; PubChem
	**Coumarins**										
34.	Dihydroxycoumarin-hexoside	15.49	C_15_H_16_O_9_	339.0710 ^a^	**177.0194**	√		√			Fragmentation; PubChem
35.	Dihydroxycoumarin	21.50	C_9_H_6_O_4_	177.0216 ^a^	159.8905; **133.0291**; 105.0344	√		√			Fragmentation; PubChem
	**Flavonoids**										
36.	Rutoside	23.15	C_27_H_30_O_16_	609.1636 ^a^	301.0132; **300.0227**; 271.0230; 255.0163; 178.9974; 151.0001	√		√	√	√	[47]
37.	Tetrahydroxymethoxyflavone *O*-rutinoside (Isorhamnetin 3-*O*-rutinoside)	24.12	C_28_H_32_O_16_	623.1696 ^a^	315.0464; **314.0414**; 300.0198; 299.0166; 271.0204; 243.0299; 151.0022	√		√			Fragmentation; PubChem
38.	Isoquercitrin	24.27	C_21_H_20_O_12_	463.0666 ^a^	**301.0368**; 300.0295; 271.0262; 255.0310; 178.9994; 151.0041	√		√			[47]
39.	Tetrahydroxyflavone-7-*O*-hexoside (Kaempferol-7-*O*-hexoside)	25.08	C_21_H_20_O_11_	447.0915 ^a^	285.0390; **284.0326**; 255.0282; 227.0343; 151.0023	√		√			Fragmentation; PubChem
40.	Tetrahydroxyflavone-3-*O*-hexoside-pentoside (Kaempferol-3-*O*-hexoside-pentoside)	25.37	C_27_H_30_O_15_	593.1599 ^a^	**285.0408**; 255.0309; 227.0366; 151.0021	√		√			Fragmentation; PubChem
41.	Pentahydroxyflavone-3-*O*-rhamnoside (Quercetin-3-*O*-rhamnoside isomer)	25.77	C_27_H_30_O_16_	609.1543 ^a^	301.0343; **300.0268**; 271.0230; 255.0306; 178.9971; 151.0054	√		√			Fragmentation; PubChem
42.	Isorhamnetin-3-*O*-hexoside	25.92	C_22_H_22_O_12_	477.1025 ^a^	**314.0428**; 299.0217; 271.0269; 151.0017	√		√			[59]
43.	Quercetin	30.63	C_23_H_20_O_12_	301.0396 ^a^	178.9975; **151.0023**	√					[47]
44.	Isorhamnetin	34.58	C_16_H_12_O_7_	315.0553 ^a^	**300.0290**; 271.0196; 151.0034	√					[59]
	**Isoprenoids**										
45.	Norisoprenoid glucoside	15.52	C_19_H_34_O_9_	451.2207 ^c^	405.2145; 225.1456; 179.0584; 167.1064	√	√	√		√	PubChem
	**Fatty acids**										
46.	4,10-dimethyl-9-[3,4,5-trihydroxy-6-(hydroxymethyl)oxan-2-yl]oxydodeca-2,4,6-trienedioic acid	25.58	C_20_H_30_O_10_	429.1831 ^a^	249.1131; 205.1232	√	√	√	√	√	PubChem
47.	Fatty acid	33.73	C_18_H_34_O_5_	329.2386 ^a^	229.1441; 211.1336; 171.1014	√	√	√	√	√	PubChem
48.	Fatty acid	46.86	C_18_H_30_O_3_	293.2158 ^a^	**275.2018**; 224.1411; 195.1386; 171.1016	√	√	√	√	√	PubChem
49.	Glyceryl linolenate	51.61	C_21_H_36_O_4_	353.2712 ^b^	335.2530; 261.2168; 243.2060	√					PubChem
50.	2-hydroxy-6-[(8*Z*,11*Z*)-pentadeca-8,11,14-trienyl]benzoic acid	51.80	C_22_H_30_O_3_	241.2119 ^a^	297.2194; 229.1177; 159.0807; 106.0422	√					PubChem
51.	Linoleic acid amide = 9,12-Octadecadienamide	52.36	C_18_H_33_NO	280.2652 ^b^	263.2340; 245.2235	√	√		√	√	PubChem
52.	Linolenic acid (9*Z*,12*Z*,15*Z*)-octadeca-9,12,15-trienoic acid	52.71	C_18_H_30_O_2_	279.2336 ^b^	109.1001; 95.0849; 81.0697; 67.0544;55.0547	√	√				PubChem
53.	Hexadecanamide	53.46	C_16_H_33_NO	256.2652 ^b^	102.0903; 88.0751; 74.0598; 57.0703	√	√	√	√	√	PubChem
54.	Oleamide	53.701	C_18_H_35_NO	282.2809 ^b^	265.2488; 248.2385	√	√		√	√	PubChem
55.	Linolenyl alcohol	54.01	C_18_H_32_O	265.2546 ^b^	247.2389	√					PubChem

M, methanol; W, water, HAE, homogenizer assisted extraction, MAC, maceration; INF, infusion.; ^a^ [M−H]^−^, ^b^ [M+H]^+^, ^c^ [M+HCOO]^−^, ions with the highest intensity in MS/MS are indicated in bold, √—compound present in the extract.

A few fatty acids (**46**, **47**, **48**, **49**, **50**, **51**, **52**, **53**, **54**, **55**) and one isoprenoid (**45**) were tentatively identified in negative or positive ionization mode.

### 3.3. Antioxidant Activity

Free radicals are responsible for oxidative damage of biomolecules in living organisms and hence triggering pathologies like Alzheimer’s, cardiovascular disease, diabetes, and cancer among others. Antioxidants prevent and limit the destructive effects of free radicles. Natural antioxidants are widely used for many functional food and pharmaceutical formulations [60]. In the present study, the antioxidant activity of different extracts of *G. acutidentatum* aerial parts was evaluated, and the results are presented in Table 3. Generally, the antioxidant activity of different extracts varied according to the solvent/method of extraction and assay. The anti-DPPH and anti-ABTS activities were in the range of 0.86–41.42 and 66.45–103.59 mg TE/g, respectively. Both methanolic extracts (HAE-M and MAC-M = 41.42 and 33.20 mg TE/g, respectively, *p* < 0.05) revealed higher anti-DPPH than all aqueous extracts (0.86–19.48 mg TE/g). Contrary to DPPH scavenging activity, the three aqueous extracts exerted significantly (*p* < 0.05) higher anti-ABTS activity than the methanolic ones, with the highest significant (*p* < 0.05) value recorded from INF-W. Nevertheless, although the two methanolic extracts had the lowest values, they exerted higher anti-ABTS activity than the anti-DPPH one. All extracts displayed considerable ion-reducing capacity, and the highest significant (*p* < 0.05) Cu^2+^ reducing capacity was obtained respectively from HAE-M (113.55 mg TE/g) and MAC-M (104.07 mg TE/g). The former also exhibited significantly (*p* < 0.05) the highest Fe^3+^ reducing capacity (74.52 mg TE/g) followed by HAE-W, INF-W, and MAC-M (65.54–60.58 mg TE/g, *p* ≥ 0.05). The chelating properties of different extracts ranged between not active and 19.81 mg EDTAE/g, and although HAE-W revealed significantly (*p* < 0.05) the highest activity, the other two aqueous extracts (MAC-W and INF-W) were not active. The highest total antioxidant activity via the phosphomolybdenum assay was shown from the two methanolic extracts (HAE-M = 1.20 and MAC-M = 1.00 mmol TE/g, *p* < 0.05).

The present study represented the first report on the antioxidant activity of *G. acutidentatum*. Overall, all extracts displayed considerable antioxidant activity, which varied according to assay and type of the extract. Although secondary metabolites are present in all plant organs, their nature, quantity, and biological potential vary according to many factors like organ studied, extraction methods, and solvent used. In the present study, it was noted that HAE by methanol allowed higher extraction of antioxidants. The HAE is an ecologically friendly and economical extraction technique as it requires a lower consumption of solvent, and the time needed for extraction is relatively short [61]. Also, hot water extraction is more suitable for recovering extracts with radical scavenger and metal-reducing properties than maceration by water. In fact, high extraction temperature for the appropriate time resulted in aqueous extracts with high antioxidant activity [10]. Antioxidant molecules like ferulic acid derivatives [62], malic acid [63], and citric acid [64] were identified in all extracts. The alkaloid boldine, detected in all extracts, was also reported to exert significant antioxidant activity [65]. Additionally, it was reported that the antioxidant potential of *p*-coumaric acid increases significantly upon conjugation with quinic acid, monosaccharides, and amines [66]. Thus, the presence of 3-*p*-coumaroylquinic acid and beta-D-glucosyl-2-coumarate in many extracts may also enhance their antioxidant activity. Furthermore, several studies reported a positive correlation between the antioxidant activity of extracts to their phenolic composition and concentration [67,68]. This agrees well with the present results obtained from the anti-DPPH, Cu^2+^ reducing, and total antioxidant activities, where the methanolic extracts revealed the highest TPC, TFC, and antioxidant activity. In addition, antioxidant molecules like quercetin, isorhamnetin and their derivatives [69,70], derivatives of kaempferol [71], and dihydroxycoumarin and its glycoside [72], which were only identified in the methanolic extracts might be responsible for its high antioxidant activity. Furthermore, the higher anti-ABTS and Fe^3+^-reducing properties of the aqueous extracts could be attributed to the presence of antioxidant compounds that are highly soluble in water. In fact, the chemical structure of molecules creates variations in their solubility properties in different solvents and, consequently, in the antioxidant activity of extracts [73]. Taken together, the obtained antioxidant results can reflect the importance of *G. acutidentatum* extracts as a valuable source of natural antioxidants, and we strongly recommend further analysis of the individual components of this plant to detect in vivo systems.

### 3.4. Enzyme Inhibition Activity

Enzymes are currently receiving increased attention due to their potential therapeutic effects for several diseases like Alzheimer’s disease, diabetes, and some skin disorders. The present study evaluated different extracts of *G. acutidentatum* aerial parts for their capacity to inhibit the AChE, BChE, Tyr, *α*-amylase, and *α*-glucosidase enzymes. Results are shown in Table 4. The two methanolic extracts (HAE-M and MAC-M) revealed significantly (*p* < 0.05) higher enzyme inhibition properties in all tested enzymes than the aqueous extracts. HAE-M recorded the highest anti-AChE (2.55 mg GALAE/g), and α-amylase inhibition (0.51 mmol ACAE/g) activities, while MAC-M exerted the best anti-BChE (3.76 mg GALAE/g) activity. They also displayed comparable anti-Tyr activity (25.15 and 26.79 mg KAE/g, *p* ≥ 0.05). All aqueous extracts were either less active or ineffective against these enzymes. The results of acetylcholinesterase inhibition activity supported those of Kocanci, Hamamcioglu and Aslım [4], who found that the activity of the methanolic extract exceeded that of the aqueous extract, and it was in a concentration-dependent manner. Furthermore, this activity could be partly attributed to caffeoylmalic acid [47] and quercetin [74], which were detected only in the methanolic extracts and were reported to exert significant anti-AChE activity. Also, many alkaloids in *Glaucium* species are proven to have neuroprotective effects. Glaucine [7] and protopine [75,76], detected in all extracts, were reported to exert significant anti-AChE activity. Dolanbay, Kocanci, and Aslim [17] reported that alkaloids of *G. corniculatum*, like α-allocryptopine, suppress oxidative stress-induced neuronal apoptosis by suppressing the mitochondrial apoptotic pathway and regulating the cell cycle. The high anti-Tyr [77] and α-amylase inhibition [78] activities of the two methanolic extracts could be associated with the richness of these extracts in flavonoids. It is worth mentioning that this is the first report on the butyrylcholinesterase, tyrosinase, and α-amylase inhibitory properties of *G. acutidentatum*, and although these tests were in vitro, the results shed some light on the activity of extracts in the direction of neuroprotection, melanogenesis, and antidiabetic effects indicated that the plant could be a promising source of enzyme inhibitors.

### 3.5. Cytotoxic Effects

**Figure 1:**
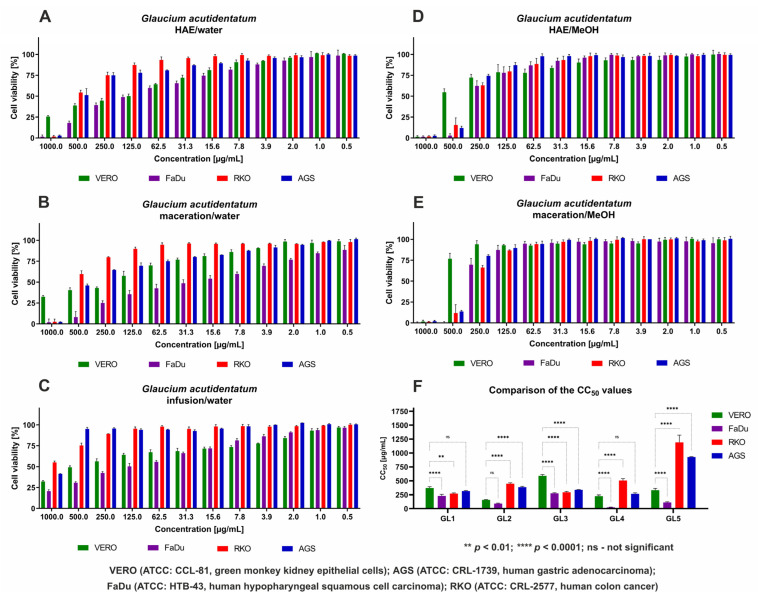
The dose–response influence of *Glaucium acutidentatum* extracts on cell lines (Dose-response effect of extracts obtained from *G. acutidentatum* using homogenizer-assisted extraction with water (**A**), maceration with water (**B**), infusion (**C**), homogenizer-assisted extraction with methanol (**D**), and maceration with methanol (**E**), (**F**) Comparison of the CC_50_ values obtained for different *G. acutidentatum* extracts).

It was observed that methanolic extracts from *G. acutidentatum* showed lower cytotoxicity toward non-cancerous cells than aqueous extracts (Table 5). Interestingly, HAE-M resulted in higher cytotoxicity (CC_50_ = 371.95 μg/mL) than maceration (CC_50_ = 591.60 μg/mL). A similar effect was observed for extraction with water; the HAE produced an extract with the highest cytotoxicity to VERO cells, with CC_50_ of 157.73 μg/mL, whereas in the case of maceration and infusion, the cytotoxicity was noticeably lower, with CC_50_ of 225.70 and 332.97 μg/mL, respectively. *G. acutidentatum* aqueous extracts did not show any selective cytotoxicity toward AGS and RKO cancer cells, while methanolic extracts showed noticeable selectivity, with the Selectivity Index (SI) between 1.17 and 2.01. MAC-M provided extracts with significantly (*p* < 0.0001) higher cytotoxicity toward RKO and AGS cells, compared to their effect toward non-cancerous cells (Figure 1F), while HAE-M showed significantly (*p* < 0.01) higher cytotoxicity only against RKO. All tested *G. acutidentatum* extracts showed selective toxicity toward FaDu cancer cells, with SI between 1.62 and 9.04. For the aqueous extract obtained by maceration, the obtained CC_50_ value (24.98 μg/mL) against FaDu was the lowest among all tested samples and was also significantly (*p* < 0.0001) lower than that obtained against non-cancerous cells indicative of its high anticancer activity (SI = 9.04). The dose–response influence of both methanolic extracts on cancer cell lines was comparable (Figure 1D,E). Conversely, aqueous extracts showed diverse effects on both the normal and cancer-originating cell lines. Both HAE and infusion-obtained aqueous extracts showed a similar effect on RKO and AGS (Figure 1A,C), but in the case of maceration-derived aqueous extract (Figure 1B), lower toxicity was observed toward RKO, compared to AGS. Figure 1B also depicts the selective toxicity of maceration-derived aqueous extract toward FaDu cells, where at the concentration of 2 μg/mL, the viability of VERO, RKO, and AGS cells was comparable to the non-treated control cells (approx. 100% viability), while the viability of FaDu cells was approx. 75%.

Kocanci et al. [4] studied the anti-proliferative potential of *G. acutidentatum* methanolic and aqueous extracts and reported the lack of cytotoxicity toward non-cancerous PC12 cells (CRL-1721; derived from a transplantable rat pheochromocytoma) and low toxicity toward cancer-originating HT-29 (human colorectal adenocarcinoma) and HeLa (human cervical adenocarcinoma) cell lines. The CC_50_ values for PC12 cells after 24 incubation with methanolic or aqueous extracts were 979 μg/mL and 1383 μg/mL, respectively. Unfortunately, the authors evaluated the anticancer potential using only two concentrations, 500 and 1000 μg/mL, and reported only the percentage of cellular viability at those concentrations, which resulted in the inability to calculate the CC_50_ values. Since higher cytotoxicity was observed toward cells originating from colorectal adenocarcinoma, in our studies, we have selected the RKO cells originating from colon cancer, as well as FaDu and AGS cells, originating from hypopharyngeal and stomach cancer, respectively.

According to the American National Cancer Institute (NCI), the criteria for significant anticancer activity for crude extracts is CC_50_ < 20 μg/mL after 48 h or 72 h incubation [79]. Other reports [80,81,82] consider CC_50_ of up to 30 μg/mL as a promising crude extract for further research. Taking this into account, it can be concluded that the MAC-W of *G. acutidentatum* shows promising anticancer activity against cells originating from human hypopharyngeal carcinoma. This activity is highly dependent on the type of cells and was not observed on RKO and AGS cells. Furthermore, some of the identified compounds in different extracts were reported to possess significant antitumor activity. For example, boldine exerted a cytotoxic effect in a concentration-dependent manner on human colorectal cancer (CRC) and osteosarcoma cell lines [83]. Isocorydine exhibited a significant anticancer effect against oral squamous cell carcinoma (OSCC) and also inhibited the proliferation of oral tongue squamous cells (Cal-27) by causing mitochondrial dysfunction and interrupting cellular energy [84]. The aporphine alkaloids, glaucine, and corydine, were shown to have inhibitory activity against several mouse tumor cell lines, including leukemia P388 and L1210, melanoma B16, bladder cancer MBC2, and colon cancer Colon 26 in culture [85]. Protopine was shown to be effective against different cancer cells like colon cancer (HCT116 and SW480), liver cancer (HepG2, HepG2, and Huh-7), breast Cancer (MDA-MB-231), pancreatic cancer (MIA Paca-2 and PANC-1), prostate cancer (HRPC), lung cancer (A549) [86]. Besides these alkaloids, rutin (flavonoid) has been reported to counteract numerous cancers via several mechanisms such as cell cycle arrest, inflammation, malignant cell growth inhibition, oxidative stress, apoptosis induction, and angiogenesis modulation, and all of these are mediated through the regulation of cellular signaling pathways [87]. Ferulic acid derivatives (phenolic acids) were shown to possess an important regularity effect on tumor resistance [88]. Future work should focus on identifying these compounds and elucidating their specific roles against the tested cell lines.

### 3.6. Molecular Docking

The binding propensity of the dominant compounds in the extracts of *G. acutidentatum* from Turkey flora against the studied target proteins was predicted using molecular docking. In general, members of the genus *Glaucium* are known to be rich in alkaloids. Therefore, we focused on the interactions between alkaloids and the selected targets. After chemical identification and based on Table 2, we selected alkaloids present in all methanol extracts because methanol extracts were generally more active than water extracts.

**Figure 2:**
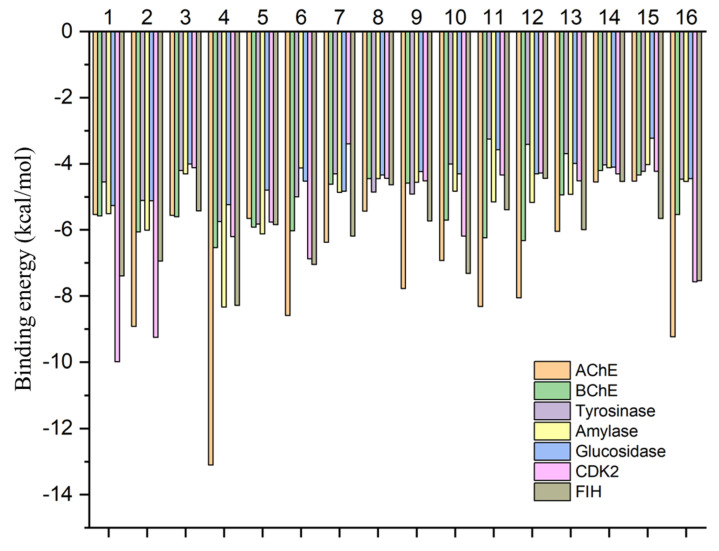
Docking score (predicted binding energy) of the main compounds in the extracts of *Glaucium acutidentatum.* (1-Lotusine, 2-Isoboldine or boldine, 3-N-methylcoclaurine, 4-1,2-dehydroreticuline, 5-Reticuline, 6-4′-O-Methyl-N-methylcoclaurine, 7-Isocorydine, 8-Laudanine, 9-Corydine, 10-3-Hydroxyglaucine, 11-Protopine, 12-a-Allocryptopine, 13-Glaucine 14-syn. Boldine dimethyl ether, 15-Cataline, 16-Corunnine).

**Figure 3:**
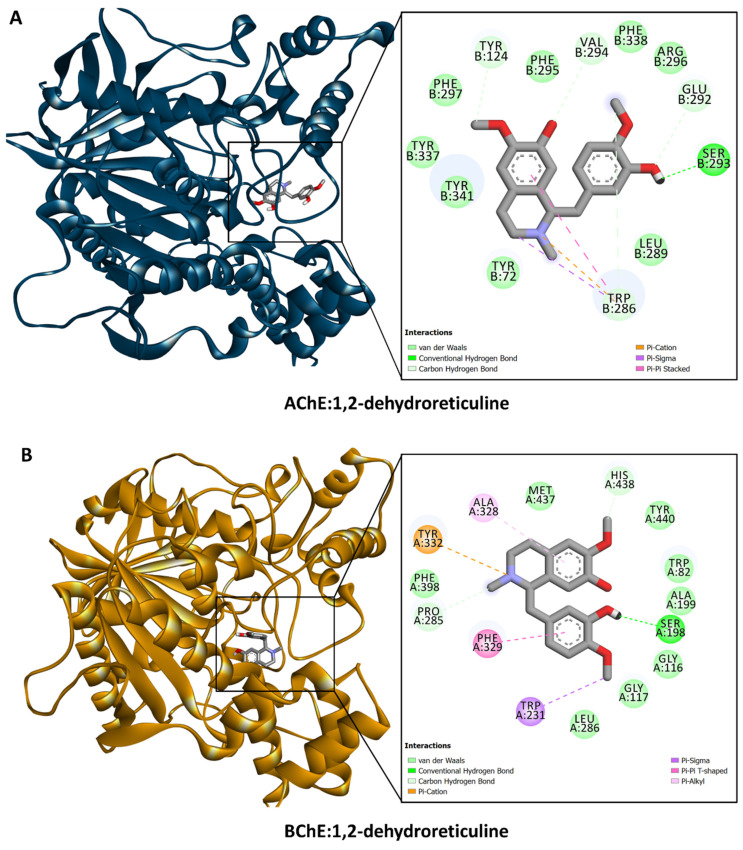
Interaction of 1,2-dehydroreticuline with (**A**) AChE and (**B**) BChE.

**Figure 4:**
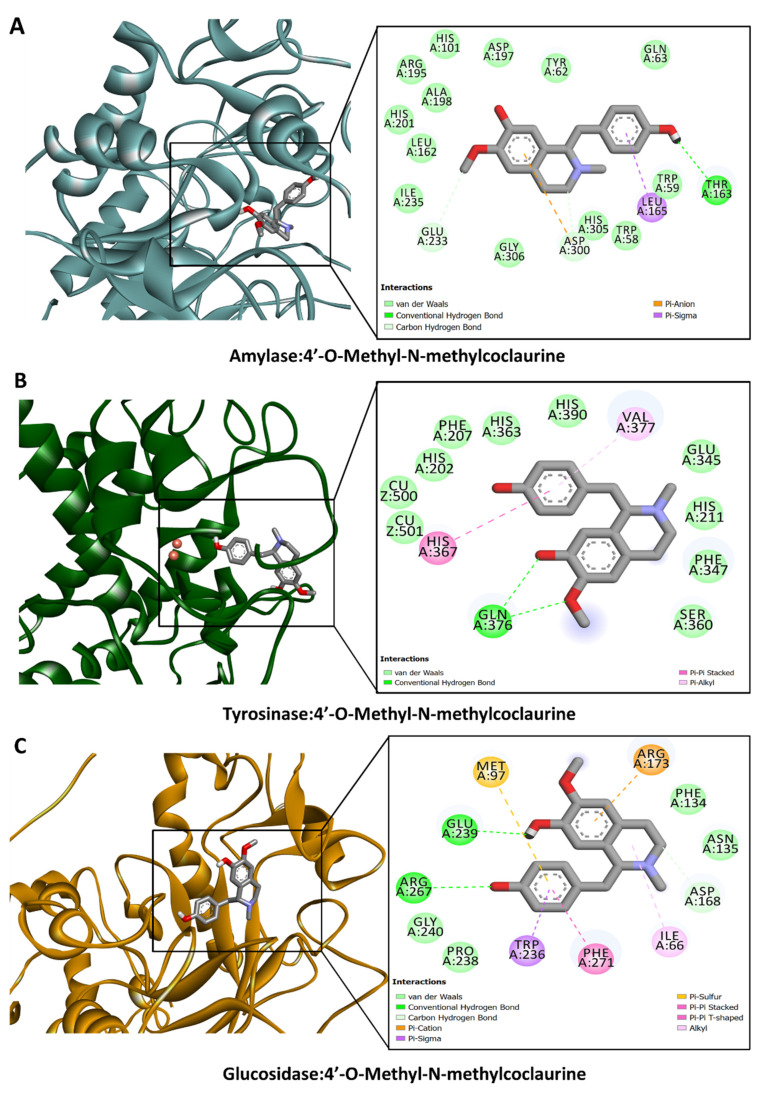
Protein–ligand interaction of 4′-*O*-Methyl-*N*-methylcoclaurine with (**A**) amylase, (**B**) tyrosinase, and (**C**) glucosidase.

The calculated binding energy scores (in kcal/mol) are shown in Figure 2. All the dominant compounds demonstrated potential binding to these target proteins, with a preference for AChE, CDK2, and FIH. For instance, 1,2-dehydroreticuline occupied the catalytic channel of AChE, forming an H-bond with Ser293, π–cation, π–sigma, and π–π stacking interactions with Trp286, as well as multiple van der Waals interactions with amino acid residues in the tunnel (Figure 3A). Trp286 is one of the important residues for the AChE activity [36]. Similarly, 1,2-dehydroreticuline formed similar interactions with the structurally related enzyme BChE: H-bond with Ser198, π–cation, π–sigma, π–π stacking, and hydrophobic interactions with Tyr332, Trp231, Phe329, and Ala328, respectively, and a few van der Waals interactions (Figure 3B). Interestingly, the binding of 4′-O-Methyl-N-methylcoclaurine to amylase (Figure 4A), tyrosinase (Figure 4B), and glucosidase (Figure 4C) was achieved through the formation of H-bonds, π–cation, π–sigma, π–π stacking, and van der Waals interactions all over the active sites of these enzymes. The catalytically essential Cu^2+^ ions in tyrosinase were also engaged in van der Waals interaction (Figure 4B).

**Figure 5:**
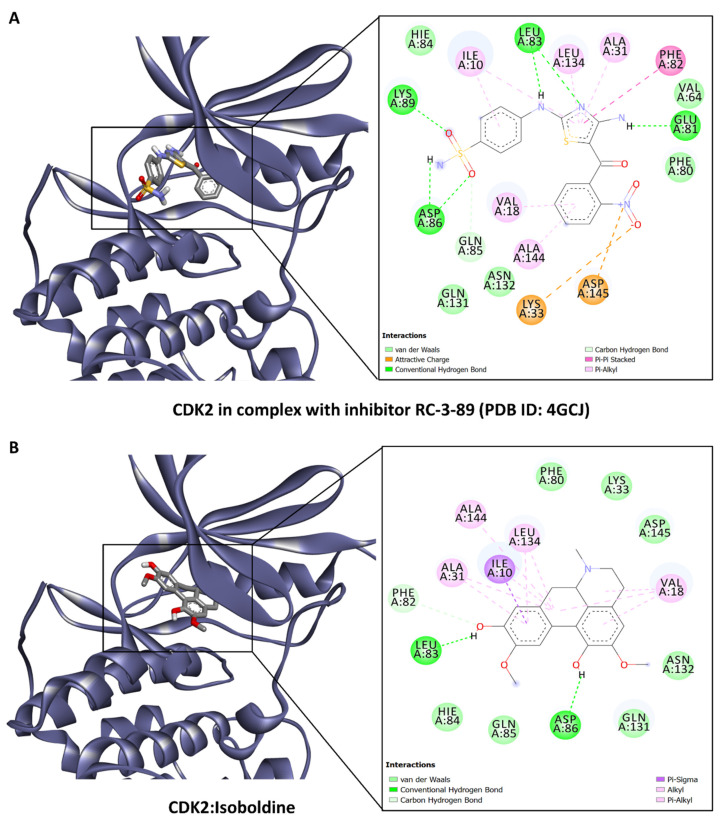
(**A**) CDK2 in complex with inhibitor RC-3-89 (PDB ID: 4GCJ) and (**B**) docking complex of CDK2 with isoboldine.

**Figure 6:**
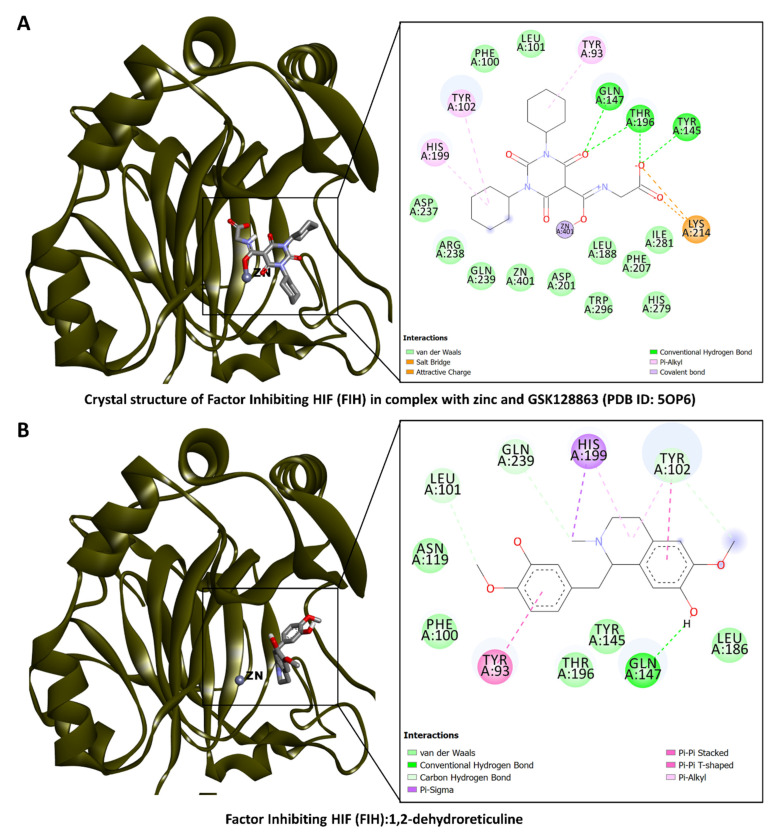
(**A**) Factor Inhibiting HIF (FIH) in complex with zinc and “GSK128863” (PDB ID: 5OP6) and (**B**) docking complex of FIH with 1,2-dehydroreticuline.

Furthermore, analysis of the binding of these bioactive compounds to possible anticancer target proteins CDK2 and FIH was carried out as described above. Molecular docking results suggested that some of the compounds, which include lotusine, isoboldine, 1,2-dehydroreticuline, 4′-*O*-Methyl-*N*-methylcoclaurine, and corunnine possess CDK2 and HIF-1 inhibitory activities. For example, of the different interactions present in the crystal structure of CDK complexed with inhibitor RC-3-89 (PDB ID: 4GCJ) (Figure 5A), an H-bond with the backbone of Leu83 and the side chain of Asp86, and hydrophobic interactions with Val18, Leu134, and Ala144 were found to be in common with the docking complex of CDK2 with isoboldine (Figure 5B). Similarly, the FIH in complex with zinc and “GSK128863” (PDB ID: 5OP6) (Figure 6A) and the docking complex of FIH with 1,2-dehydroreticuline were compared (Figure 6B). Among the interacting residues found in common, even though in some cases, the interaction was not the same, were Tyr93, Phe100, Tyr102, Gln147, His199, and Thr196 via different interactions, including H–bonds, π–cation, π–sigma, π–π stacking, and hydrophobic interactions (Figure 6). Collectively, these interactions may add up to block the activity of the proteins. Molecular docking is intended to provide initial insights into the interactions between the components and ligands. Since the matrix of plant extracts is very complex and the possible interactions between them (antagonistic or synergistic) are very complex, the abilities cannot be explained only by the presence of a single compound. Therefore, the isolation of the selected compounds and their biological activities is strongly recommended in future studies.

## 4. Conclusions

The present study presented the first detailed report on the phytoconstituents, antioxidant, cytotoxic, and enzyme inhibition capacity of *G. acutidentatum.* The study also demonstrated the impact of solvents and techniques of extraction on biological activities. Results indicated that the aerial parts contained aporphine, beznyltetrahydroisoquinoline, and protopine types of alkaloids and were rich in phenolics. Different extracts exerted variable antioxidant and enzyme inhibition activities. However, using methanol as a solvent in homogenizer-assisted extraction recovered substances with the highest antiradical (DPPH) and ions-reducing capacity as well as enzyme inhibition activity against all tested enzymes. Substances with the best anti-ABTS and chelating properties were extracted by water through infusion and homogenizer-assisted extraction, respectively. Thus, it was clear that *G. acutidentatum* is a promising source of alkaloids and phenolic compounds for variable pharmaceutical formulations. Quantification of the identified compounds is recommended to provide insight into the relative abundance of these substances in different extracts. Also, the isolation of bioactive molecules, using effective and environmentally friendly methods, and illustration of their mechanism of action and safety, as well as in vivo studies, are warranted.

## Figures and Tables

**Table 1:** Total phenolic and flavonoid contents in extracts from *Glaucium acutidentatum* aerial parts.

Extract	TPC (mg GAE/g)	TFC (mg RE/g)
HAE-M	53.22 ± 0.10 ^a^	20.30 ± 0.25 ^b^
HAE-W	23.10 ± 0.16 ^e^	2.10 ± 0.14 ^c^
MAC-M	36.49 ± 0.05 ^b^	30.28 ± 0.51 ^a^
MAC-W	23.81 ± 0.16 ^d^	1.32 ± 0.47 ^c^
INF-W	30.88 ± 0.43 ^c^	1.94 ± 0.38 ^c^

Values are reported as mean ± SD of three parallel measurements. GAE: Gallic acid equivalents; RE: Rutin equivalents. M, methanol; W, water, HAE, homogenizer assisted extraction, MAC, maceration; INF, infusion. Different letters in the same column indicate significant differences in the extracts (*p* < 0.05).

**Table 3:** Antioxidant properties of extracts from *Glaucium acutidentatum* aerial parts.

Extracts	DPPH (mg TE/g)	ABTS (mg TE/g)	CUPRAC (mg TE/g)	FRAP (mg TE/g)	Chelating (mg EDTAE/g)	PBD (mmol TE/g)
HAE-M	41.42 ± 0.62 ^a^	77.00 ± 2.01 ^c^	113.55 ± 6.44 ^a^	74.52 ± 4.74 ^a^	15.42 ± 0.33 ^b^	1.20 ± 0.17 ^a^
HAE-W	8.94 ± 0.32 ^d^	89.52 ± 0.43 ^b^	60.96 ± 1.65 ^d^	65.54 ± 3.15 ^b^	19.81 ± 0.05 ^a^	0.03 ± 0.01 ^d^
MAC-M	33.20 ± 0.27 ^b^	66.45 ± 5.79 ^d^	104.07 ± 1.04 ^b^	60.58 ± 0.40 ^b^	12.79 ± 0.29 ^c^	1.00 ± 0.12 ^b^
MAC-W	0.86 ± 0.03 ^e^	91.70 ± 0.89 ^b^	61.78 ± 0.35 ^d^	42.60 ± 1.03 ^c^	na	0.06 ± 0.01 ^d^
INF-W	19.48 ± 0.48 ^c^	103.59 ± 1.49 ^a^	85.76 ± 0.47 ^c^	65.13 ± 2.05 ^b^	na	0.40 ± 0.03 ^c^

Values are reported as mean ± SD of three parallel measurements. PBD: Phosphomolybdenum; MCA: Metal chelating Activity; TE: Trolox Equivalent; EDTAE: EDTA equivalent. M, methanol; W, water, HAE, homogenizer assisted extraction, MAC, maceration; INF, infusion. Different letters in the same column indicate significant differences in the extracts (*p* < 0.05).

**Table 4:** Enzyme inhibitory properties of in extracts from *Glaucium acutidentatum* aerial parts.

Extracts	AchE (mg ALAE/g)	BChE (mg GALAE/g)	Tyrosinase (mg KAE/g)	Amylase (mmol ACAE/g)
HAE-M	2.55 ± 0.10 ^a^	1.45 ± 0.10 ^b^	25.15 ± 1.00 ^a^	0.51 ± 0.02 ^a^
HAE-W	1.25 ± 0.09 ^c^	1.09 ± 0.03 ^c^	na	0.09 ± 0.01 ^d^
MAC-M	2.07 ± 0.11 ^b^	3.76 ± 0.31 ^a^	26.79 ± 2.36 ^a^	0.45 ± 0.01 ^b^
MAC-W	0.65 ± 0.06 ^d^	na	na	0.30 ± 0.01 ^c^
INF-W	0.53 ± 0.05 ^d^	na	na	0.09 ± 0.01 ^d^

Values are reported as mean ± SD of three parallel measurements. GALAE: Galantamine equivalent; KAE: Kojic acid equivalent; ACAE: Acarbose equivalent; na: not active. M, methanol; W, water, HAE, homogenizer assisted extraction, MAC, maceration; INF, infusion. Different letters in the same column indicate significant differences in the extracts (*p* < 0.05).

**Table 5:** Cytotoxicity and anticancer selectivity of *Glaucium acutidentatum* extracts.

*Glaucium acutidentatum*	VERO	FaDu		AGS		RKO	
	CC_50_	CC_50_	SI	CC_50_	SI	CC_50_	SI
HAE-M	371.95 ± 22.69	229.60 ± 28.08	1.62	317.50 ± 8.84	1.17	270.45 ± 12.29	1.38
HAE-W	157.73 ± 7.12	90.95 ± 9.17	1.73	387.17 ± 16.2	0.41	447.87 ± 15.97	0.35
MAC-M	591.60 ± 21.45	274.65 ± 11.70	2.15	337.25 ± 6.72	1.75	294.70 ± 16.78	2.01
MAC-W	225.70 ± 20.95	24.98 ± 4.78	9.04	266.20 ± 17.88	0.85	506.73 ± 32.68	0.45
INF-W	332.97 ± 32.17	112.13 ± 11.75	2.97	929.40 ± 69.93	0.36	1191.50 ± 129.40	0.28

CC_50_—50% cytotoxic concentration (μg/mL ± SD), calculated from at least three replicates; HAE—homogenizer-assisted extraction; M, methanol; W, water; MAC—maceration; INF—infusion; SI—anticancer selectivity index (CC_50_VERO/CC_50_Cancer cells).

## Data Availability

All data and materials used or generated in this study are available and may be provided on request by the corresponding author.

## Supplementary Materials

The following supporting information can be downloaded at: https://www.mdpi.com/article/10.3390/antiox13060643/s1.
